# Draft Genome Sequence of a New *Methanobacterium* Strain Potentially Resistant to Bile Salts, Isolated from Deer Feces

**DOI:** 10.1128/MRA.00548-20

**Published:** 2020-08-06

**Authors:** Heleen T. Ouboter, Stefanie Berger, Erbil Güngör, Mike S. M. Jetten, Cornelia U. Welte

**Affiliations:** a Department of Microbiology, Institute for Water and Wetland Research, Radboud University, Nijmegen, The Netherlands; University of Maryland School of Medicine

## Abstract

We present the high-quality draft genome of Methanobacterium subterraneum DF, a hydrogenotrophic methanogen that was isolated from deer feces. This organism has potentially been overlooked in previous studies. Interestingly, its genome encoded bile salt hydrolase, a crucial enzyme for bile salt tolerance that is found in gut organisms.

## ANNOUNCEMENT

Methanogens are well-known inhabitants of the microbiome, producing the potent greenhouse gas methane. They may also play a role in the pathogenesis of several intestinal disorders and may be related to energy metabolism and adipose disposition (1, 2). In this study, DNA was extracted from an anoxic methanogenic culture originating from deer feces. The cultures were incubated in Hungate tubes at 37°C at 150 rpm. The medium contained yeast extract (0.1 g/liter), Casitone (0.1 g/liter), NaCl (0.5 g/liter), NH_4_Cl (0.5 g/liter), KH_2_PO_4_ (0.15 g/liter), MgCl_2_·6H_2_O (0.3 g/liter), CaCl_2_·2H_2_O (0.1 g/liter), NaHCO_3_ (2.5 g/liter), resazurin (1 mg/liter), vitamins as described for DSMZ medium 141 (1 ml/liter), and trace elements (1 ml/liter), as described previously (3). The medium was sparged with N_2_/CO_2_ (80:20) and autoclaved, and Na_2_S/cysteine-HCl (0.5 g/liter) and 50 mM trimethylamine were added together with H_2_/CO_2_ (80:20) at 100-kPa overpressure. Active methane-producing samples were subcultured. DNA could be isolated only after vigorous bead beating for 10 min using the PowerSoil DNA extraction kit (MO BIO Laboratories, Uden, Netherlands). For library preparation, the Nextera XT library kit (Illumina, San Diego, CA, USA) was used. The library was normalized to 4 nM and sequenced with an Illumina MiSeq system using the 300-bp paired-end protocol. Default parameters were used for all software unless otherwise specified. Trimming and assembly were performed with CLC Genomics Workbench (v20.0; Qiagen Aarhus). Sequencing data were trimmed using default settings with subsequent removal of 40 bp at the 5′ side. This yielded 7,328,633 paired-end reads with an average length of 194.4 bp and an average genome coverage of 41.4×. Reads were assembled *de novo* (word size, 22; maximum bubble size, 203; minimum contig length, 1,000; length fraction, 0.5; similarity fraction, 0.95). Contigs were binned using RStudio (v1.2.5019) (4) plotting GC content versus sequencing depth, which yielded a draft genome composed of 40 contigs with a mean contig length of 59,324 bp and an *N*_50_ value of 100,222 bp. The genome was assessed using CheckM (v1.0.11), which yielded 93.0% completeness and 2.8% contamination (5). Genes were annotated using PGAP (v4.11) (6), and the KEGG Automatic Annotation Server (KAAS) (7) was used to check for relevant pathways. BLASTp (https://blast.ncbi.nlm.nih.gov/Blast.cgi) was used to manually curate the annotations. The genome size was 2,372,968 bp, with a GC content of 39.8%, and was composed of 2,313 predicted genes. Using average nucleotide identity (ANI), the newly enriched methanogen had 99.1% similarity to Methanobacterium subterraneum A8p (GenBank accession number NZ_CP017768.1), which is found in deep subterranean granitic aquifers (8), and 87% similarity to Methanobacterium formicicum BRM9 (BioProject number PRJNA49593). The ANI values were calculated using an online calculator (http://enve-omics.ce.gatech.edu/ani) with default settings (minimum length, 700 bp; minimum identity, 70%; minimum alignment, 50; window size, 1,000 bp; step size, 200 bp). The new strain was named Methanobacterium subterraneum DF. In agreement with previous work, M. subterraneum DF was a hydrogenotrophic methanogen, using CO_2_ and H_2_ or formate to produce CH_4_ (8). All genes required for methanogenesis, the pentose phosphate pathway, DNA/RNA precursors, gluconeogenesis, the mevalonate pathway, and the reductive tricarboxylic acid cycle were found. Genes for acetyl-coenzyme A synthetase (*acs*) and an acetate transporter were found, indicating acetate as a potential alternative carbon source. Genes for the production of most amino acids were present. Genes encoding transporters for Ca^2+^, Mg^2+^, Fe^2+^, WO_4_^2−^, PO_4_^3−^, and K^+^ were present. Ammonium was the main nitrogen source, and sulfide was the main sulfur source. Analogous to the gut methanogens Methanobrevibacter smithii and Methanosphaera stadtmanae, a gene encoding bile salt hydrolase (BSH) was found in the genome of M. subterraneum DF (9). BSH facilitates survival of microorganisms in the gut by increasing their bile salt tolerance (10), indicating that this novel strain of M. subterraneum may be a genuine inhabitant of the deer intestinal tract. Due to the very difficult DNA retrieval, this new strain might have been overlooked in previous studies and could be an important gut microbe.

### Data availability.

This whole-genome shotgun project has been deposited in DDBJ/ENA/GenBank under the accession number JABBYL000000000; the raw reads are available under accession number SRP261944. The version described in this paper is version JABBYL010000000.
